# Isolation and characterization of severe acute respiratory syndrome coronavirus 2 in Turkey

**DOI:** 10.1371/journal.pone.0238614

**Published:** 2020-09-16

**Authors:** Shaikh Terkis Islam Pavel, Hazel Yetiskin, Gunsu Aydin, Can Holyavkin, Muhammet Ali Uygut, Zehra Bestepe Dursun, İlhami Celik, Ceren Cevik, Aykut Ozdarendeli

**Affiliations:** 1 Department of Microbiology, Medical Faculty, Erciyes University, Kayseri, Turkey; 2 Vaccine Research, Development and Application Center, Erciyes University, Kayseri, Turkey; 3 Gen Era Diagnostics Inc. Barbaros, Istanbul, Turkey; 4 Department of Microbiology and Infectious Disease, Kayseri City Training and Research Hospital, Kayseri, Turkey; University of Tennessee Health Science Center, UNITED STATES

## Abstract

Coronavirus disease 2019 (COVID-19) caused by severe acute respiratory syndrome coronavirus 2 (SARS-CoV-2) and associated with severe respiratory illness emerged in Wuhan, China, in late 2019. The virus has been able to spread promptly across all continents in the world. The current pandemic has posed a great threat to public health concern and safety. Currently, there are no specific treatments or licensed vaccines available for COVID-19. We isolated SARS-CoV-2 from the nasopharyngeal sample of a patient in Turkey with confirmed COVID-19. We determined that the Vero E6 and MA-104 cell lines are suitable for supporting SARS-CoV-2 that supports viral replication, development of cytopathic effect (CPE) and subsequent cell death. Phylogenetic analyses of the whole genome sequences showed that the hCoV-19/Turkey/ERAGEM-001/2020 strain clustered with the strains primarily from Australia, Canada, England, Iran and Kuwait and that the cases in the nearby clusters were reported to have travel history to Iran and to share the common unique nucleotide substitutions.

## Introduction

Coronaviruses (CoVs) are members of the family Coronaviridae, which consists of a group of enveloped, positive-sense, single-stranded RNA viruses [1]. Transcription of coronaviruses requires a polymerase template switch, characterized as a discontinuous process unique among RNA viruses [2–4]. Based on the difference in protein sequences, CoVs are classified into four genera, alpha-CoV, beta-CoV, gamma-CoV, and delta-CoV2 [1, 2, 5]. There are hundreds of coronaviruses are circulating broadly among mammals and birds that cause respiratory, enteric, hepatic, and neurologic diseases [1, 6, 7].

Until recently, six coronavirus species have been known to cause disease in humans. The 229E, OC43, NL63 and HKU1 viruses are prevalent and cause mild illness, such as the common cold [1, 8]. However, the other two viruses have been considered highly pathogenic in humans, and cause the diseases SARS (severe acute respiratory syndrome), which resulted from an outbreak in 2002 and disappeared by 2004, and MERS (Middle East respiratory syndrome), which emerged in 2012 and continues to circulate in the Middle East [9–13]. At the end of 2019, severe pneumonia cases of unknown etiology were reported in Wuhan, a city in the Hubei province of China [14–15]. Sequencing analysis revealed that this unidentified pneumonia was considered to be caused by a novel coronavirus [14, 16]. The World Health Organization (WHO) termed the disease as coronavirus disease-2019 (COVID-19) on February 11, 2020 [17]. On the same day, the International Committee on Taxonomy of Viruses (ICTV) named this novel coronavirus as severe acute respiratory syndrome coronavirus 2 (SARS-CoV-2). SARS-CoV-2 has become the seventh coronavirus that known to infect humans.

Even though the first cases had a contact history with the Huanan Seafood Market the studies have clearly showed that SARS-CoV-2 can be transmitted by person-to-person and frequently cause asymptomatic infections. With transmission of the virus possible before the onset of clinical signs, the COVID-19 outbreak has quickly expanded to worldwide [18–20]. It was declared a pandemic by the WHO on March 11, 2020. As of August 18, 2020, a total of 21,756,357 confirmed cases of COVID-19 and 771,635 deaths have been reported in more than 200 countries and territories. (https://www.who.int/emergencies/diseases/novel-coronavirus-2019/situation-reports/). The first case of COVID-19 in Turkey was confirmed on March 112020. As of August 18, 2020, there have been 251,805 cases and 6,016 deaths (The Ministry of Health, Turkey).

In this study, we report the isolation of the hCoV-19/Turkey/ERAGEM-001/2020 strain from a patient in Turkey with confirmed COVID-19. The whole genomic sequence and replication characteristics of the hCoV-19/Turkey/ERAGEM-001/2020 strain are described. This is the first known report of the isolation and characterization of SARS-CoV-2 from a human clinical sample in Turkey. The successful isolation and characterization of the virus will be essential for continued investigations of SARS-CoV-2 pathogenicity and will provide valuable information for vaccine design and drug target.

## Materials and methods

### Ethics statement

This study protocol was approved by the Kayseri Training and Research Hospital ethics committee (2020-3-/23), which allowed sampling for diagnostic and surveillance purposes. A written informed consent was obtained from the patient for being included in the study.

### Cells

All cell lines used in this study purchased from ATCC cell culture company. African monkey green kidney cells (Vero E6, ATCC CRL-1586), rhesus monkey kidney cells (MA-104, ATCC CRL-2378), human adrenal carcinoma cells (SW-13, ATCC CCL-105) and human cervical adenocarcinoma cells (HeLa, ATCC CCL-2) were cultured in Dulbecco’s modified Eagle’s medium (DMEM) (Sigma, Germany) supplemented with 10% heat-inactivated fetal bovine serum (FBS) (Gibco, USA), 100 mM L-glutamine, 100 U/ml penicillin, 100 μg/ml streptomycin (Biological Industries, USA). All cell lines tested were found to be free of mycoplasma using the EZ-PCR Mycoplasma Detection Kit (Biological Industries, USA).

### Virus isolation

A patient was admitted to the Kayseri City Training and Research Hospital on March 17, 2020 due to respiratory symptoms. The patient’s nasopharyngeal sample was obtained by using a UTM^™^ kit containing 1 ml of viral transport media (Copan Diagnostics, USA) on day 4 of his illness. The diluted sample was inoculated onto monolayers of Vero E6 cells and gently agitated at 37°C for 1 h. Consequently, DMEM with 2% FBS was added and the infected cells were monitored for the appearance of cytopathic effect (CPE). All handling of the virus was conducted in a biosafety level 3 enhanced facility (BSL-3).

### First-strand cDNA synthesis and PCR

Viral RNA was isolated from 140 μl of the infected culture supernatant using the QIAamp Viral RNA Mini Kit (Qiagen, Germany) according to the manufacturer’s recommendations. The viral RNA was reverse transcribed using the Moloney murine leukemia virus reverse transcriptase (M-MLV RT) (Thermo Scientific, USA) using random hexamers according to the manufacturer’s recommendations. The reaction mixtures were incubated for 60 min at 42°C, and the reaction was stopped by heating the mixture at 95°C for 5 min and chilling it on ice. The primers used in PCR reactions were designed according to the sequences published by the Centers for Disease Control and Prevention (CDC) [21]. The PCR was conducted in a 50 μl reaction mixture containing 3 μl of cDNA template, 10 mM Tris-HCl, 50 mM KCl, 1.5 mM MgCl_2_, 2019-nCoV_N1 forward primer (5′-GACCCCAAAATCAGCGAAAT-3′), 2019-nCoV_N3 reverse primer (5′- TGTAGCACG ATT GCAGCATTG -3′), 1 U of Taq polymerase (Thermo Scientific, USA), and 1.25 mM dNTPs. The cycling conditions were 94°C for 3 min, followed by 35 cycles of 94°C for 45 sec, 55°C for 45 sec, and 72°C for 1 min with a final extension at 72°C for 10 min. The PCR products were visualized by ethidium bromide staining after 1% agarose gel electrophoresis. The PCR reactions were also set up with two different combinations of the primers under the same conditions as described above. The primers used in the PCR reactions were 2019-nCoV_N1 forward primer (5′-GACCCCAAAATCAGCGAAAT-3′), 2019-nCoV_N2 reverse primer (5’-GCGCGACATTCCGAAGAA-3’) and 2019-nCoV_N3 forward primer (5’-GGGAGCCTTGAATACACCAAAA-3’), and 2019-nCoV N2 reverse primer (5’-GCG CGACATTCCGAAGAA-3’), respectively.

### Plaque assay

Twenty-four-well plates were seeded with Vero E6 cells and incubated at 37°C with 5% CO_2_. The monolayer was inoculated with 10-fold serially dilutions of the virus. After incubation for 1 h at 37°C with shaking, the monolayer was overlaid with 0.5 ml overlay medium containing 0.3% low melting agarose (Sigma, Germany). After incubation at 37°C for 3 days, the cells were fixed with 10% formalin (v/v) for 90 min at room temperature. The agarose overlay was discarded, and the plaques were visualized by staining the monolayer with 1% crystal violet (w/v) in 20% ethanol (v/v).

### Virus titration

We cultured to SARS-CoV-2 passage 1 (1:100 dilution) in Vero E6 cells to make virus the passage 2 virus stock. The SARS-CoV-2 virus lysate was then harvested at 48 h post-infection and the supernatants were collected, clarified, and stored at -80°C. To determine the titer of the passage 2 virus a focus forming assay (FFA) was performed as described previously [22]. Briefly, Vero E6 cells were seeded on 96 well-plates and incubated at 37°C with 5% CO_2_ for overnight. The cell monolayers were inoculated with 10-fold serial dilutions virus at 2nd passage. The diluted samples were added in triplicate to confluent Vero cell monolayers. After absorption for 1 h at 37°C, the supernatants were removed and the cells were washed with PBS. The cell monolayers were overlaid with virus medium containing 1% CMC (carboxymethyl cellulose) then incubated at 37°C with 5% CO_2_ for 72 h. After fixation with 10% neutral buffered formaldehyde at room temperature for 20 min, the cells were permeabilized with 0.1% Triton X-100 in PBS for 20 min with gentle rocking and blocked with 5% skim milk in PBS. The wells were then incubated with a human antibody to SARS-CoV-2 nucleocapsid protein (1:2500) (GenScript; HC2003) for 1h in TBST (100 mM Tris–HCl pH 8.0, 1.5 M NaCl, 1% Tween 20) at 37°C and washed 3 times with TBST. The cells were incubated for 1 h with goat anti-human IgG conjugated to fluorescein isothiocyanate (FITCH) (Southern Biotech, USA) and diluted 1:1000 in TBST and then washed three times with TBST and once with distilled water. The antibody-labeled cells were detected and analyzed by immunofluorescence microscopy (Leica, UK). The fluorescent foci in each well were counted, and the virus titers were calculated and expressed as fluorescent focus units (FFU) per ml as described previously [22]. To obtain the virus passage 3 virus stock, the Vero E6 cells were infected with the virus passage 2 virus at an MOI of 0.01, and the viral lysate were was harvested at 48 h post-infection and the supernatants were collected. Subsequently, the virus passage 4 virus was generated in Vero E-6 cells infected with virus passage 3 virus at an MOI of 0.01.

### Protein analysis and Western blot

Cell lysates were harvested in Laemmli sodium dodecyl sulfate–polyacrylamide (SDS) gel electrophoresis sample buffer containing 2% SDS and 5% β-mercaptoethanol. The lysates were boiled and loaded onto a polyacrylamide gel. The samples were separated on 12% resolving and 5% stacking SDS-PAGE gels in a mini electrophoresis unit (Bio-Rad, USA) at 100 V for 1 h. The proteins were transferred onto a nitrocellulose membrane (Millipore, USA) under wet conditions using a trans-blot apparatus (Bio-Rad, USA). After blocking with 5% skimmed milk, the membrane was incubated either with a rabbit polyclonal to SARS-CoV-2 spike glycoprotein (1/1000) (Abcam; ab272504) or a human antibody to the SARS-CoV-2 nucleocapsid protein (1:2500) (GenScript; HC2003) followed by a goat anti-rabbit horseradish peroxidase (HRP)-conjugated antibody (1:2000 dilution, Invitrogen; USA) and a goat anti-human horseradish peroxidase (HRP)-conjugated antibody (1:2000 dilution, Invitrogen; USA), respectively. B-actin was used as a loading control in Western blot. The membrane was reacted with the ECL substrate solution (Pierce ECL, USA). The membrane was exposed to an autoradiograph film (KODAK X-OMAT, Sigma Germany), and was developed using a Kodak developer (X-OMAT 1000A, Sigma Germany).

### Viral replication kinetics

Vero E-6 and MA-104 cells cultured in 24-well-plates were infected with an MOI at an 0.1 (passage 4 virus). The cultures were harvested by scraping cell monolayers from at different time points (6, 12, 18, 24, 48 and 72 h) and stored at -80°C. The Vero E-6 cells were then inoculated with 10-fold serial dilutions of the samples in triplicate per dilution. The viral inoculum was removed. The cell monolayers were overlaid with virus medium containing 1% CMC. The cells were fixed with 10% neutral buffered formaldehyde after infection of 72 h at room temperature for 20 min, and permeabilized with Triton X-100. The wells were then incubated with a human antibodt to the SARS-CoV-2 nucleocapsid protein (1:2500) (GenScript; HC2003) for 1h in TBST at 37°C and washed 3 times with TBST. The cells were incubated for 1 h with goat anti-human IgG conjugated to fluorescein isothiocyanate (FITCH) (Southern Biotech, USA) and diluted 1:1000 in TBST and then washed three times with TBST and once with distilled water. Antibody-labeled cells were detected and analyzed by immunofluorescence microscopy (Leica, UK). The fluorescent foci in each well were counted, and the virus titers were calculated and expressed as fluorescent focus units per ml.

### Whole genome sequencing

For whole genome sequencing of hCoV-19/Turkey/ERAGEM-001/2020, Vero E6 cells infected with the virus were used for RNA extraction. The RNA was extracted by using the QIAamp Viral RNA Mini Kit (Qiagen, Germany). The viral RNA was reverse transcribed by M-MLV RT using random hexamers according to the manufacturer’s recommendations.

The 26 DNA amplicons the from full genome amplification [23] were quantified using the Quant-it dsDNA HS Assay Kit (Invitrogen, USA) and pooled in equal concentrations. The libraries were prepared using pooled amplicons with Nextera DNA Flex Library Prep Kit (Illumina, San Diego, CA) and sequenced on an Illumina Nextseq 500 (Illumina, USA) platform with a 2x150 Cycle Kit (Gen Era Diagnostics Inc., Turkey). The quality of the raw data was evaluated by FastQC v.0.11.5 (Babraham Bioinformatics) and low-quality bases, primers and remnant adapters were trimmed using Trimmomatic v.0.32 [24]. The reads were aligned to the previously assembled sequence of the SARS-CoV-2 genome (GenBank Accession: MN908947.3) using the Burrows-Wheeler aligner v.0.7.1 with the MEM algorithm [25]. The variants were called by using Genome Analysis Toolkit (GATK) v.3.8.0 with the HaplotypeCaller algorithm [26] and were manually inspected in GenomeBrowse v2.1.2 (GoldenHelix). The variants that had low quality and a low variant fraction (%<60) were filtered. The filtered variants and reference SARS-CoV-2 genome were used to generate the consensus sequence using bcftools v1.9 [27].

For multiple sequence alignment, complete (>29,000bp) and high-coverage genomes (n = 3970) were used from the GISAID database. The GISAID strain genomes including the genome of our strain were aligned using the MAFFT v7.450 tool [28]. Phylogenetic analysis of the alignment was performed using the IQ-Tree v. 1.6.12 with a general time-reversible (GTR) model [29]. The whole genome sequence was submitted to GenBank (ID:MT327745.1) and GISAID (ID: EPI_ISL_424366) and the raw data deposited on SRA (SAMN15062833).

### Statistical analysis

GraphPad prism 7 software (GraphPad, USA) was used to perform all statistical analysis and graphics. Mann-Whitney U test was used to find significant differences between viral passages. The significance level was set as a *p* value of less than 0.05 where *p<0.05.

## Results

### Isolation of hCoV-19/Turkey/ERAGEM-001/2020 from a human nasopharyngeal sample

**Fig 1:**
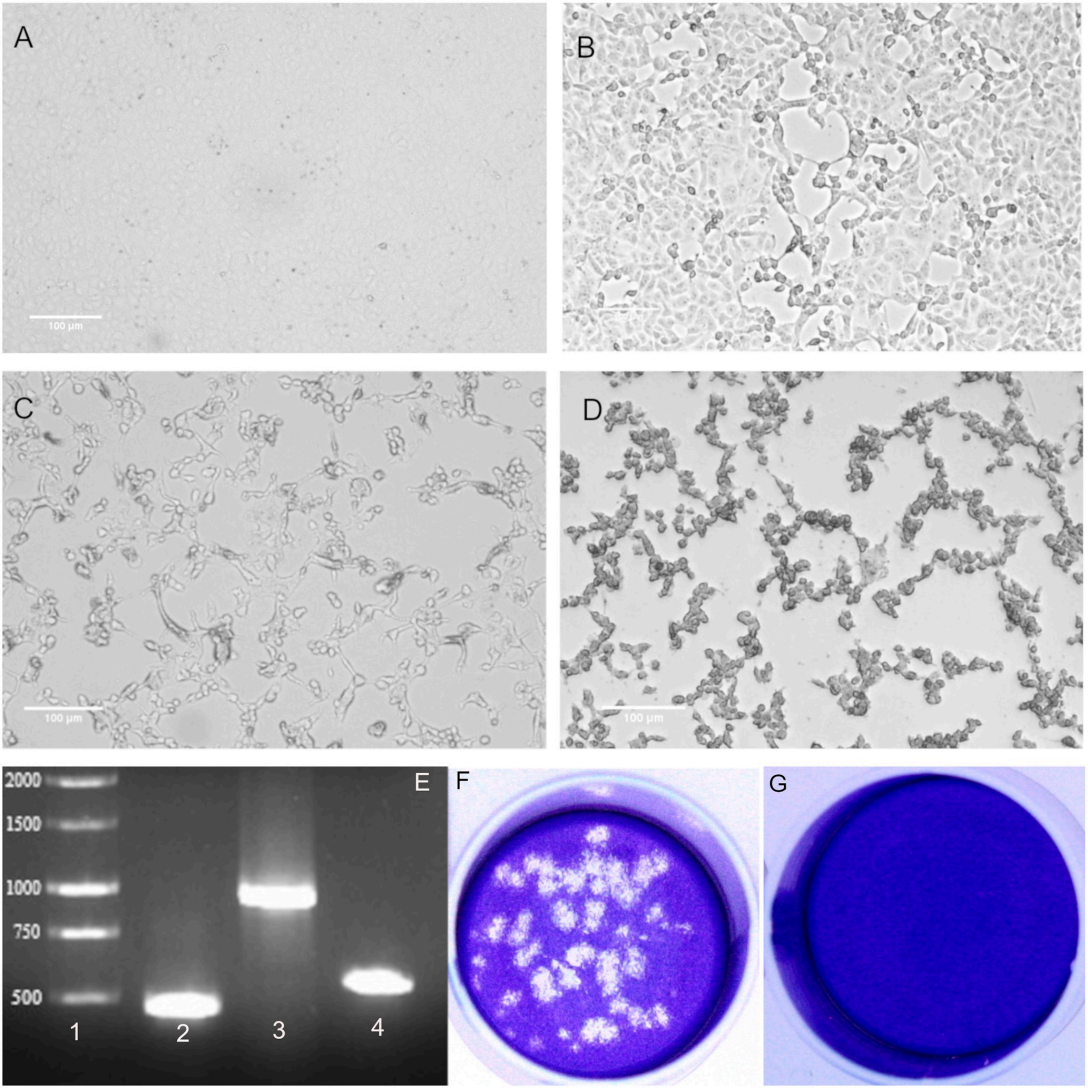
Isolation of hCoV-19/Turkey/ERAGEM-001/2020 in Vero E6 cells from the nasopharyngeal sample of a patient with COVID-19 in Turkey in 2020. Phase contrast microscopy (Leica, DMi1) of culture containing (A) mock-infected Vero E6 cells, (B) Vero E6 cells at 24 h post-infection, (C) Vero E6 cells at 48 h post-infection (D), and Vero E6 cells at 72 h post-infection. Scale bars = 100 μm. (E) RT-PCR amplification of viral RNA from Vero E6 cells infected with hCoV-19/Turkey/ERAGEM-001/2020 (1% gel). (1) Molecular weight marker 1 kb (MW). Target amplicons: (2) SARS-CoV-2 NP1 amplified with 2019-nCoV_N1 forward and 2019-nCoV_N3 reverse primers, 469 bp. (3) SARS-CoV-2 NP2 amplified with 2019-nCoV_N1 forward and 2019-nCoV_N2 reverse primers, 945 bp, and (4) SARS-CoV-2 NP3 amplified with2019-nCoV_N3 forward 2019-nCoV and 2019-nCoV N2 reverse primers, 549 bp, 1% gel. Plaque morphology for SARS-CoV-2 on Vero E6 cells at 72 h post-infection. (F) SARS-CoV-2 stock was plaqued on Vero E6 cells and visualized by crystal violet staining. (G) An uninfected control; the dilution factor of SARS-CoV-2 used for infection was a 10^−4^.

After 24 h of incubation, very little but visible CPE was detected in virus passage 1 virus (Fig 1B). The time for the onset of CPE was typically 48 h post-infection (Fig 1C) and major CPE was observed within 72 h post-infection (Fig 1D). The cells showed some morphological changes such as cell rounding, detachment/floating and degeneration whereas no such changes were observed in the uninfected cells (Fig 1A, 1B, 1C and 1D).

As we observed CPE in the infected monolayers, RT-PCR was used as a confirmatory assay. The primer set from CDC [21] targeting the nucleocapsid protein gene (NP) of SARS-CoV-2 was used for the PCR reactions. A PCR product size of 469 bp was amplified with the 2019-nCoV_N1 forward and 2019-nCoV_N3 reverse primers (Fig 1E, lane 2). We amplified two PCR products, with sizes of 945 bp and 549 bp, with the 2019-nCoV_N1 forward, and 2019-nCoV_N2 reverse primers and with the 2019-nCoV_N3 forward and 2019-nCoV_N2 reverse primers, as in shown Fig 1E lane 3 and Fig 1E lane 4, respectively.

We also performed to plaque assay to purify of SARS-CoV-2 for subsequent use in further experiments. Representative SARS-CoV-2 plaques in the Vero E6 cell monolayers infected with SARS-CoV-2 are shown in Fig 1F and 1G. Taken together, these results suggest that a SARS-CoV-2 strain named hCoV-19/Turkey/ERAGEM-001/2020 was successfully isolated from the nasopharyngeal sample of a patient in Turkey with confirmed COVID-19.

### Virus titration

**Fig 2:**
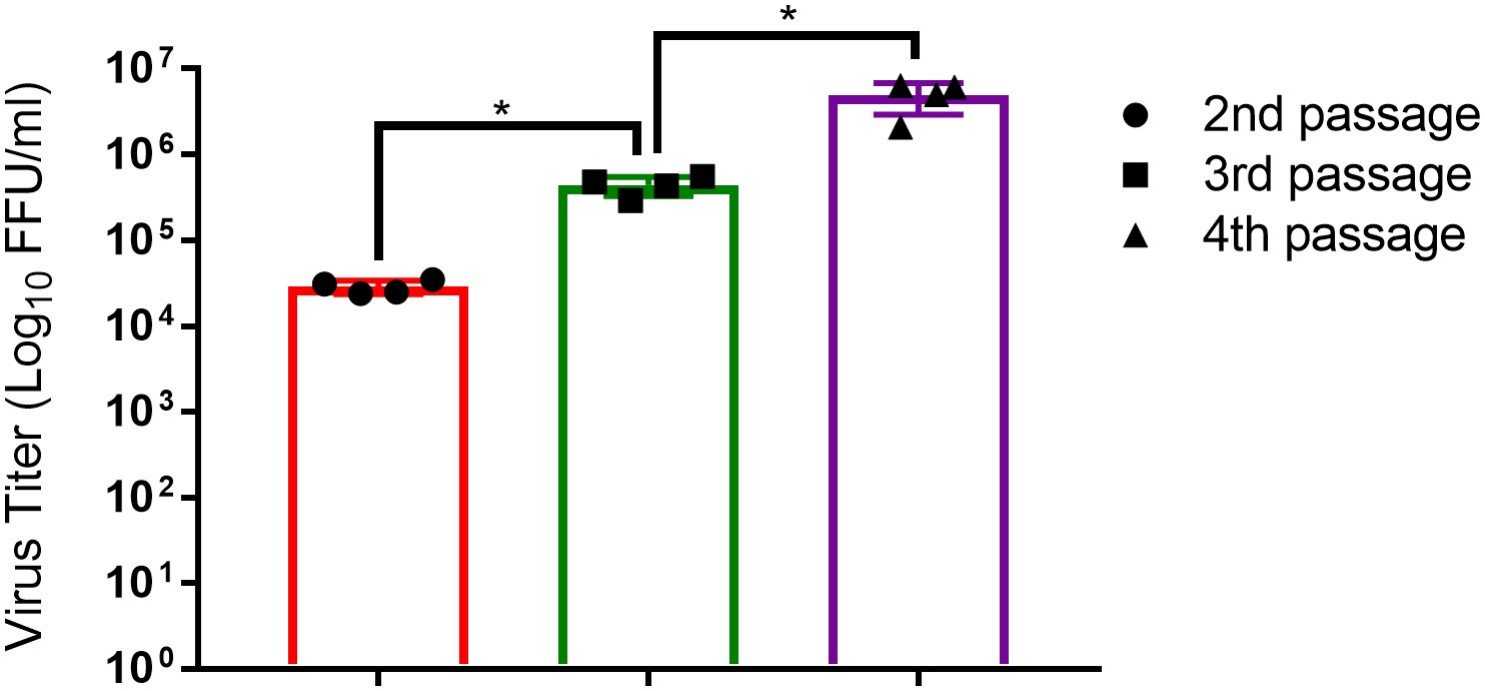
Passaging of hCoV-19/Turkey/ERAGEM-001/2020 in Vero E6 cells led to an increase in the viral titer. The viral supernatant was collected at 48 h post-infection in each passage and tested by a focus forming assay (FFA) for titration of the virus at the 2nd to the 4th passages. Fluorescent foci in each well were counted, and the virus titers were calculated and expressed as fluorescent focus units per ml (FFU/ml). A Mann-Whitney U test was performed for comparisons between different passage numbers of virus where p<0.05 considered significant, *p<0.05. The error bar represents ± the standard deviation.

We cultured to SARS-CoV-2 passage 1 in Vero E6 cells to make the virus passage 2 virus stock. Subsequently, the passage 2 virus stock was passaged two more times in Vero E6 cells. The virus stocks were quantified by using FFA (Fig 2). We determined that the titer of the passage 2 virus was 2.8x10^4^ FFU/ml, while the titers of the passage 3 and passage 4 viruses were 4.3x10^5^ FFU/ml and 4.9x10^6^ FFU/ml, respectively (Fig 2). These results indicated that propagation of the hCoV-19/Turkey/ERAGEM-001/2020 strain in Vero E6 cells led to an increasing in the viral titers in each passage.

### Permissiveness of hCoV-19/Turkey/ERAGEM-001/2020 among different cell lines

**Fig 3:**
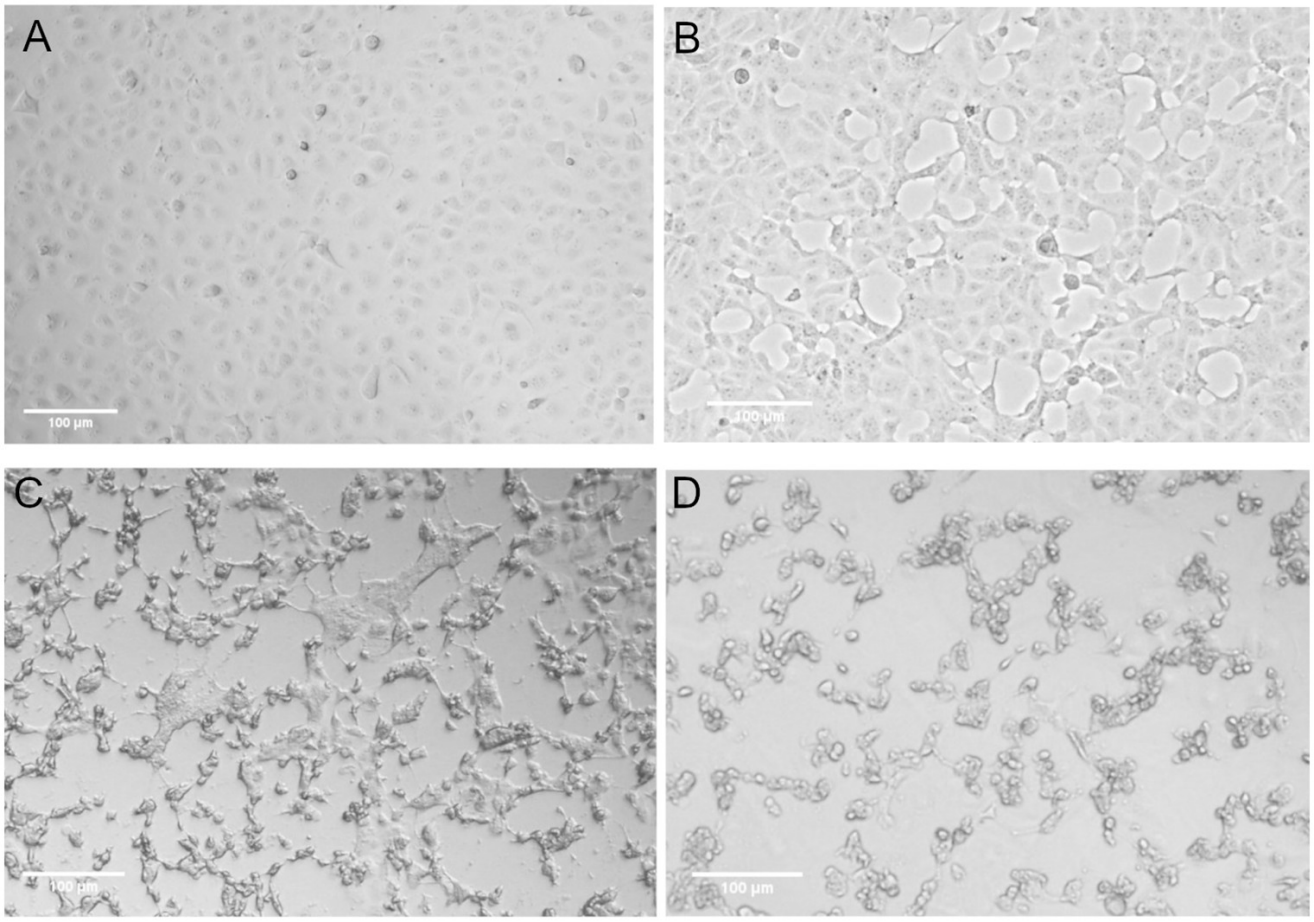
MA-104 cells supported replication of hCoV-19/Turkey/ERAGEM-001/2020. Phase contrast microscopy (Leica, DMi1) of cultures containing (A) mock-infected MA-104 cells, (B) MA-104 cells at 24 h post-infection, (C) MA-104 cells at 48 h post-infection, and (D) MA-104 cells at 72 h post-infection. Scale bars = 100 μm.

In addition to Vero E6 cells, we examined the susceptibility of MA-104, SW-13 and HeLa cell lines to infection by SARS-CoV-2. All cell lines were infected with an MOI of 0.1 (virus passage 4 virus) and monitored for CPE until 72 h post-infection. Only the MA-104 cell line developed CPE. In abnormal areas, small clusters of rounded cells, cell detachment and degeneration were observed (Fig 3). Similar to Vero E6 cells infected with SARS-CoV-2, CPE formation in the MA- infected cells began at 24 h post-infection (Fig 3B) and increased at 48 h post-infection (Fig 3C). The complete CPE was observed within 72 h post-infection (Fig 3D).

**Fig 4:**
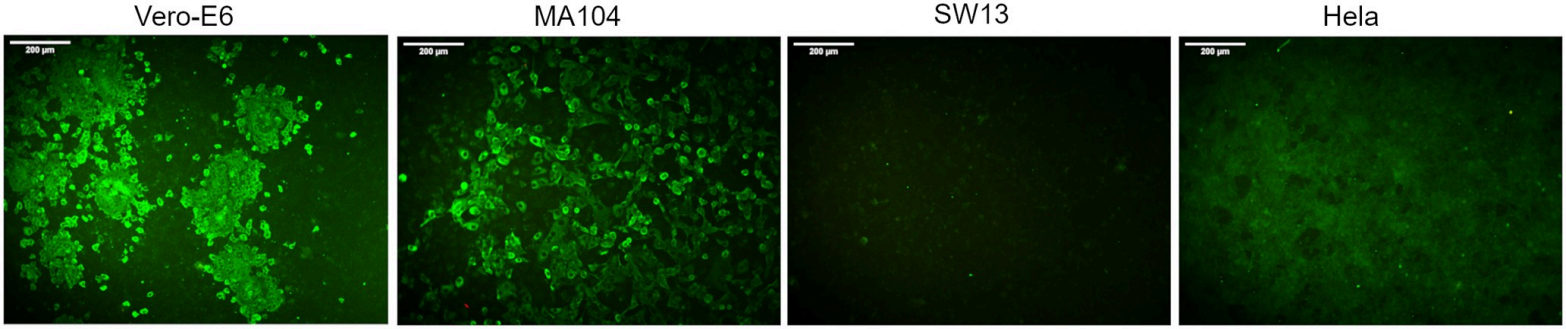
Susceptibility of different cell lines to infection with hCoV-19/Turkey/ERAGEM-001/2020. The cell lines were infected with at an MOI of 0.1, washed after adsorption, and the cell monolayers were overlaid with virus medium containing 1% CMC and incubated at 37°C with 5% CO_2_ for 48 h. After fixation with 10% formaldehyde, the cells were permeabilized with 0.1% Triton X-100 and blocked with 5% skim milk in PBS. The wells were then incubated with a human antibody to the SARS-CoV-2 nucleocapsid protein (1:2500) (GenScript; HC2003) for 1 h in TBST (100 mM Tris–HCl pH 8.0, 1.5 M NaCl, 1% Tween 20) at 37°C, followed by washing three times, after which the cells were incubated for 1 h with goat anti-human IgG conjugated to FITCH (Southern Biotech, USA). The antibody-labeled cells were detected and analyzed by immunofluorescence microscopy (Leica, DFC450C). Scale bars = 200 μm.

To confirm the results of the susceptibility of the MA-104, SW-13 and HeLa cell lines to infection by SARS-CoV-2, all cell lines were infected with an MOI of 0.1 (passage 4 virus) and incubated at 48 h post-infection. An immunofluorescence assay (IFA) confirmed that the Vero E6 and MA-104 cell lines supported the replication of SARS-CoV-2. In contrast, SARS-CoV-2 did not replicate in SW-13 and HeLa cells. (Fig 4).

**Fig 5:**
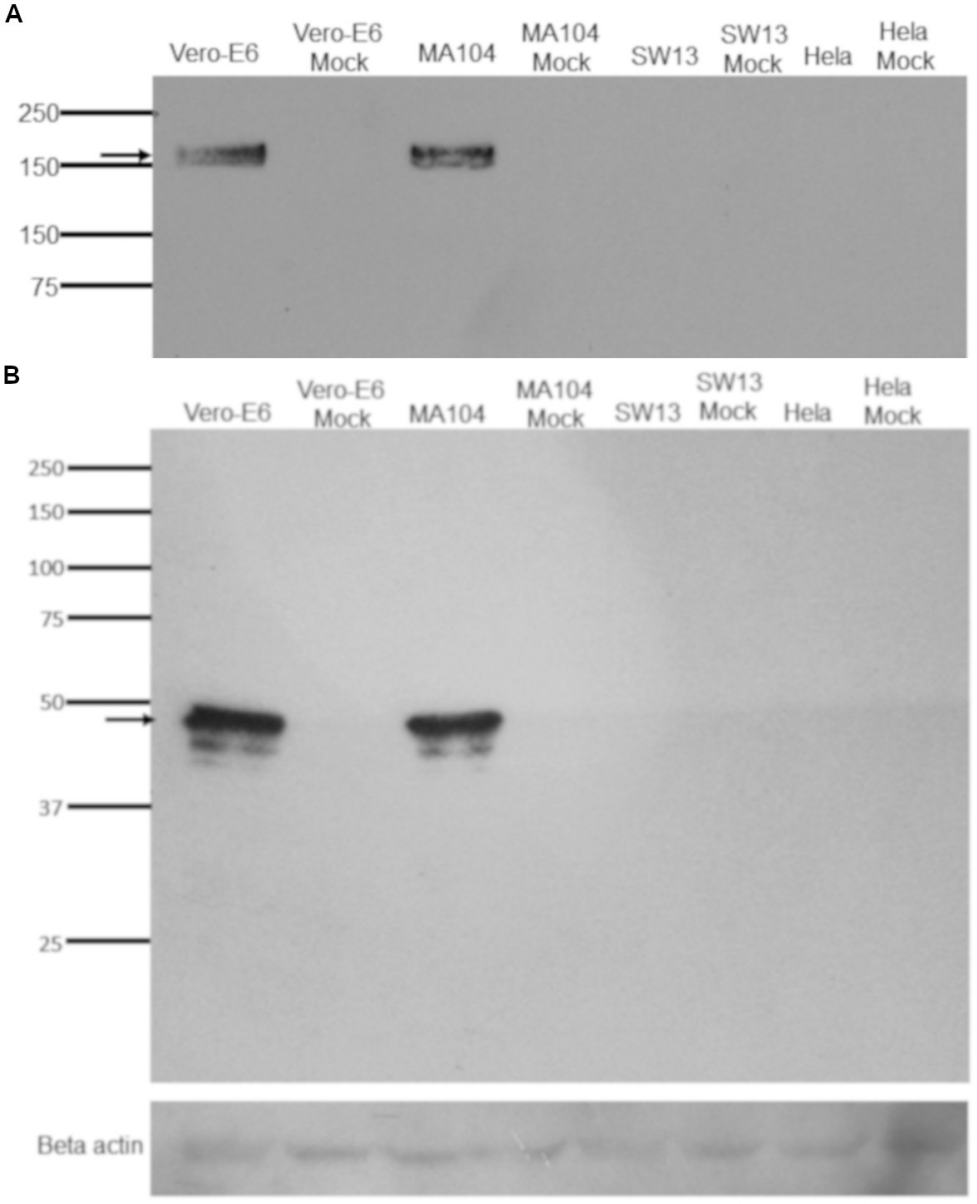
Western blot assay to examine SARS-CoV-2 proteins production. The hCoV-19/Turkey/ERAGEM-001/2020 strain was used to infect the cell lines at an MOI of 0.5. The whole cell lysate samples were collected at 24 h post-infection. The Western blot assay was performed to examine the production of viral proteins using a rabbit polyclonal antibody to the SARS-CoV-2 spike glycoprotein (S) (1/1000) (Abcam; ab272504) and a human antibody to the SARS-CoV-2 nucleocapsid protein (NP) (1:2500) (GenScript; HC2003). The membrane was reacted with the ECL substrate solution (Pierce ECL, USA). The membrane was exposed to an autoradiograph film (KODAK X-OMAT, Sigma Germany), and was developed using a Kodak developer (X-OMAT 1000A, Sigma Germany). The arrows indicate that the bands at approximately 180 kDa (Fig 5A) and 48 kDa (Fig 5B) represent S and NP, respectively.

To expand these observations, we examined the expression of the SARS-CoV-2 proteins. All cell lines were infected with an MOI of 0.5 (passage 4 virus). Cell lysates from infected cell lines were harvested at 24 h post-infection and were probed either with the rabbit polyclonal antibody to SARS-CoV-2 spike glycoprotein or with a human antibody to the SARS-CoV-2 nucleocapsid protein. SARS-CoV-2 spike protein (S) expression was detected in Vero E6 and MA-104 cell lines that supported SARS-CoV-2 replication (Fig 5A). The Vero E6 and MA-104 cell lines also showed a SARS-CoV-2 nucleoprotein (NP) band, as shown in Fig 5B. Consistent with the IFA results (Fig 4), viral antigen expression was not detectable in the nonsusceptible SW-13 and HeLa cell lines (Fig 5). Overall, these results showed that the Vero E6 and MA 104 cell lines can be efficiently infected by SARS-CoV-2.

### Viral replication kinetics

**Fig 6:**
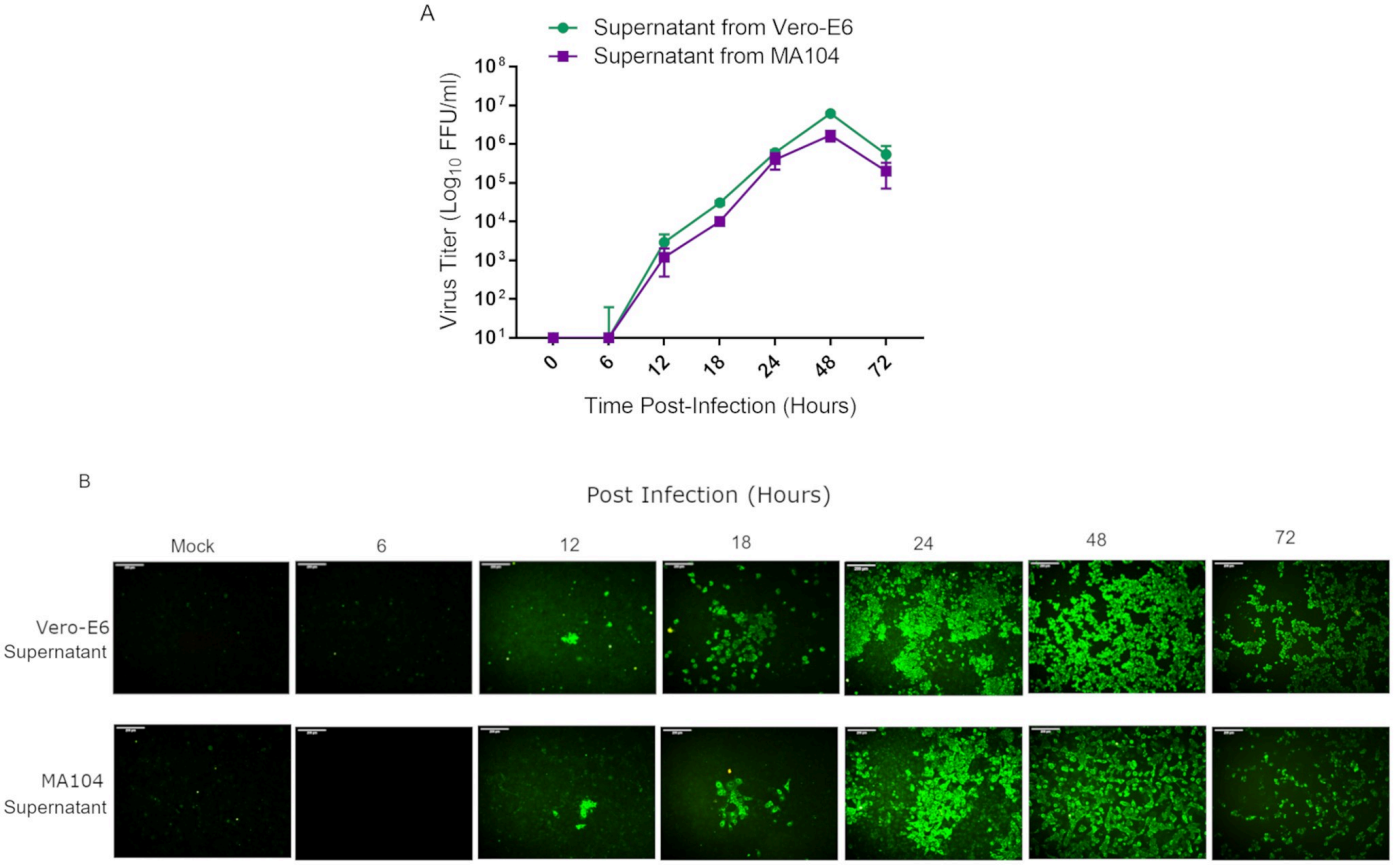
Multi-step growth kinetics of hCoV-19/Turkey/ERAGEM-001/2020. Vero E6 and MA-104 cell lines were infected at an MOI of 0.1. (A) The culture supernatants were harvested from at different time points (6, 12, 18, 24, 48 and 72 h) and stored at -80°C. The Vero E6 cells were then inoculated with 10-fold serial dilutions of the samples in triplicate per dilution. The virus titers were determined by a focus forming assay (FFA) at the indicated intervals and were assayed in triplicate. The fluorescent foci in each well were counted, and the virus titers were calculated and expressed as fluorescent focus units per ml (FFU/ml). The standard bars represent standard deviations of the mean of the results. (B) The infected cells (Vero E6 supernatant, upper panel, and MA-104 supernatant, lower panel) were stained with the FITC-labeled goat anti-human IgG at the indicated time points and examined under a fluorescence microscope (Leica, DFC450C); for each group, the representative image out of three replicates is shown. Scale bars = 200 μm.

To assess the replication kinetics, Vero E6 and MA-104 cells were infected with at an MOI of 0.1, and the supernatants were harvested at different time points (6, 12, 18, 24, 48 and 72 h post-infection). The Vero E6 and MA-104 cell monolayers were then inoculated with 10-fold serial dilutions of the samples. Viral titers of the samples were determined by FFA (Fig 6A). The growth kinetics study showed that SARS-CoV-2 replicated rapidly and efficiently and could be detected within 6 h post-infection in Vero E6 and MA-104 cells (Fig 6A and 6B). SARS-CoV-2 replicated in Vero E6 and MA-104 cells with similar kinetics and achieved similar peak titers of 6.1xlog^6^ FFU/ml and 1.6xlog^6^ FFU/ml at 48 h of post-infection, respectively (Fig 6A). However, the viral titers decreased after 48 h infection. At 72 h post-infection, the titers from the samples Vero E6 and MA-104 infected with SARS-CoV-2 were 5.4xlog10^5^ FFU/ml and 2xlog10^5^ FFU/ml, respectively (Fig 6A).

### hCoV-19/Turkey/ERAGEM-001/2020 whole genome sequencing

The sequencing produced approximately 4.9 M paired reads (150 bp x2), of which 95.2% of the reads were mapped to the reference genome. After alignment, 99.6% of the genome was covered with a 26200x sequencing depth on average. The whole-genome sequencing of hCoV-19/Turkey/ERAGEM-001/2020 revealed that the strain had 6 variants, compared to the MN908947.3 reference genome. The detected 2 non-synonymous, 3 synonymous, and 1 UTR variants are listed in Table 1. The variants were found to correspond to the genomic positions 1397 and 11083 (ORF1ab gene), 23876 (S gene), 26688 (NP gene), 29563 (ORF 10 gene) and 29742 (3’ UTR). The mutations at 1397 and 23876 are non-synonymous, leading to a change from Valine to Isoleucine in genes ORF1ab and S (Table 1).

**Table 1:** Genetic diversity of hCoV-19/Turkey/ERAGEM-001/2020 compared to the MN908947.3 reference genome of the SARS-CoV-2 Wuhan-Hu-1 isolate.

Pos	Gene	Ref	Alt	AA Pos	Ref AA	Alt AA	Depth	VF (%)
1397	ORF1ab	G	A	378	Val	Ile	45501	99.6
11083	ORF1ab	G	T	3606	Leu	Leu	17037	72.3
23876	S	G	A	772	Val	Ile	21239	99.9
28688	NP	T	C	139	Leu	Leu	35885	99.7
29563	ORF10	C	T	2	Gly	Gly	55600	99.4
29742	3'UTR	G	T	-	-	-	78352	99.7

Pos = Position, Ref = Reference, Alt = Alternative, AA = Amino Acids, VF (%) = Variant Fraction.

**Fig 7:**
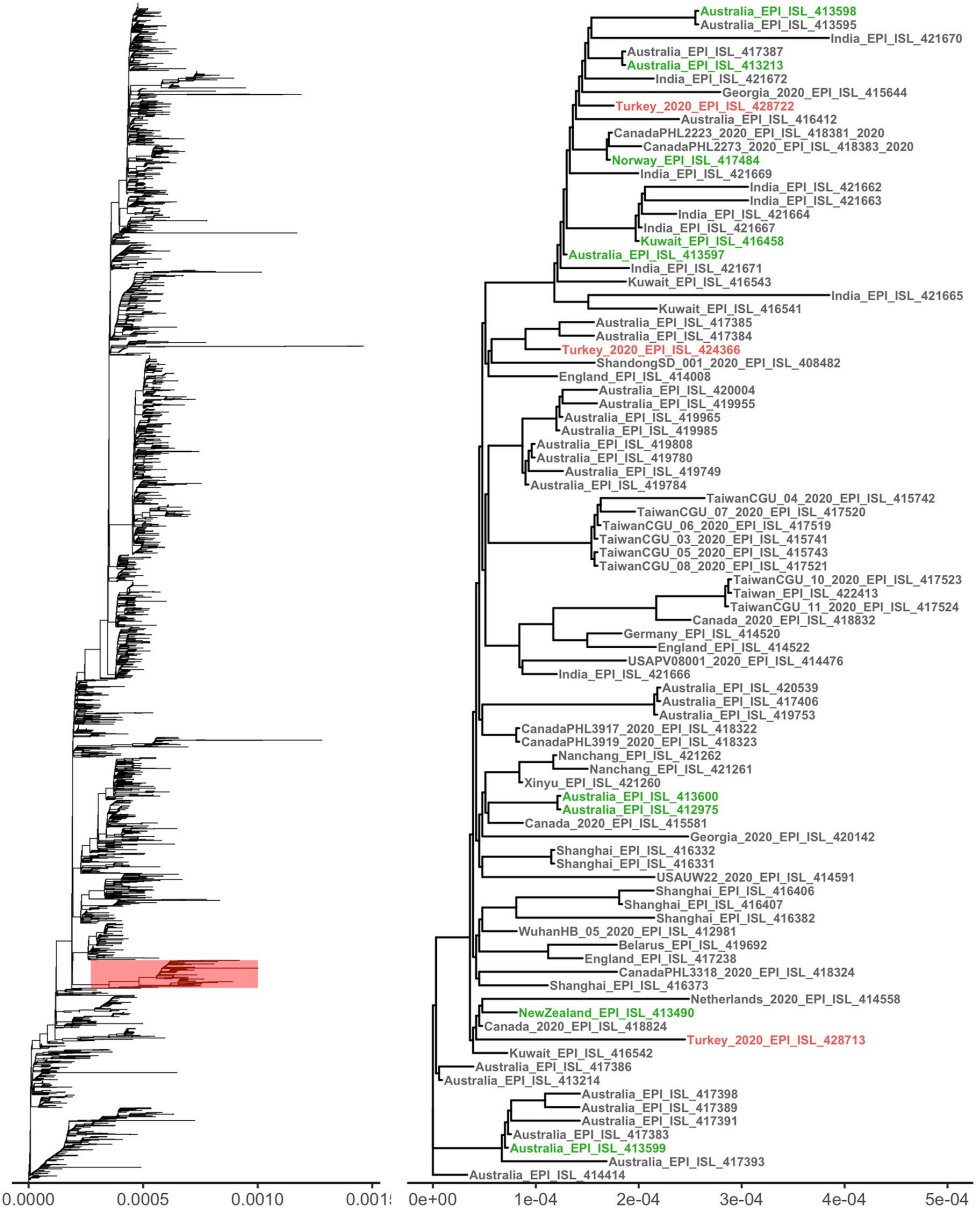
Phylogenetic analysis of the full-length genomes of SARS-CoV-2. (A) A phylogenetic tree of known sequences of SARS-CoV-2 (n = 3970) and the location of our cluster. (B) The sub-tree includes the strain of hCoV-19/Turkey/ERAGEM-001/2020, and other strains from Turkey are highlighted in red. The cases that were known to have G1397A, T28688C, and G29742T mutations and an Iran travel history or direct contact with the Iran travelers are highlighted in green.

The phylogenetic analysis showed that hCoV-19/Turkey/ERAGEM-001/2020 was located outside of the main clades (S, G, and V) and clustered with SARS-CoV-2 isolates from Australia, Canada, England and Kuwait (Fig 7). This geographically dispersed cluster is known to be branched in the early period of the epidemic and genetically clustered very closely together. Our strain shared three distinct mutations (G1397A, T28688C, and G29742T) with all members of this cluster, and those mutations were not observed in other known clusters. According to published case reports and GISAID metadata entries, at least 9 SARS-CoV-2 samples in the nearby clusters had a recent travel history to Iran [30]. Those samples in nearby clades include several cases from Pakistan, Kuwait, Canada, and Norway (Fig 7). The genome sequences of all those cases with a history of travel to Iran share three nucleotide substitutions (G1397A, T28688C, and G29742T) in the SARS-CoV-2 genome, which were also found in the hCoV-19/Turkey/ERAGEM-001/2020 strain. The GISAID had only one full genome sequence of SARS-CoV-2 from Iran, which also included the two key mutations (G1397A and G29742T). In addition, analysis of the NP partial gene sequences of the Iranian samples in GISAID showed that all of the Iranian partial sequences (n = 15) also contain the T28688C, which is another key single nucleotide polymorphism (SNP) of this clade. Our strain also contained G23876A, a non-synonymous mutation in the S gene, which was not observed in any other full genome sequences in GISAID. The mutation leads to a Val to Ile change at the 772nd position, which is located at one of the inner coil motifs of the S protein. Another mutation in the ORF10 gene (C29563T) is also quite rare and found only in two cases in Australian samples.

This study generated the second whole genome sequence of a SARS-CoV-2 strains in Turkey. The first sequence was generated in March 2020 and deposited in GISAID (EPI_ISL_417413). The first sequence was not included in the phylogenetic analysis because of its high number of unique mutations (0.44%). However, the sequence comparison showed that both sequences carry key Iranian cluster mutations including G1397A, T28688C, and G29742T. The G11083T, G23876A, and C29563T nucleotide changes in our strain were not found in the first sequenced strain in Turkey. As of April 2020, a total of 17 SARS-CoV-2 sequences have been submitted to GISAID samples from Turkey. Two of those sequences (Turkey/HGSM/5516/2020 and Turkey/HGSM/8010/2020) were located in the same cluster and share cluster-specific variants (G1397A, T28688C, G29742T). The rest of the submitted strains clustered with several other European clades.

## Discussion

SARS-CoV-2 is an emerging coronavirus that is highly infectious and efficiently transmitted through droplets and close contact. The virus has been able to spread promptly to several countries throughout the world [31–35]. The SARS-CoV-2 pandemic is likely to have serious effects on not only people’s health but also societies, health system worldwide and the global economy [36–38]. In the current outbreak situation, it is crucial to isolate of the causative virus for vaccine strain production, initial characterization of antiviral candidates and evaluation of diagnostic tools. SARS-CoV-2 was first isolated by using human airway epithelial cells on January 7, 2020 [14, 39]. Following to the first isolation of SARS-CoV-2 in China, several groups have also isolated SARS-CoV-2 by using the Vero cell line [40–43]. In this study, we isolated the SARS-CoV-2 from the nasopharyngeal sample of a patient in Turkey with confirmed COVID-19. Isolation of SARS-CoV-2 was successfully achieved in Vero E6 cells in the absence of trypsin. SARS-CoV-2 caused morphological changes such as rounding, detachment/floating and degeneration (Fig 1B, 1C and 1D).

It is essential to define different target cells for SARS-CoV-2 for further studies on virus-host interactions. Chu and colleagues identified different cell lines in which both SARS-CoV and SARS-CoV-2 replicated efficiently, but the cytopathic effects were only seen in the non-human primate kidney cell lines VeroE6 and FRhK4 [40]. Recently, a study showed that the Vero E6 and Vero CCL81 cell lines were infected with SARS-CoV-2 and exhibited to SARS-CoV-2 specific CPE. These authors found that HUH7.0 and 293T cells showed only modest viral replication but no CPE was observed, suggesting that both Vero cell types support amplification and replication of SARS-CoV-2 but that Vero E6 cells are more suitable for amplification and quantification [42]. In this study, we assessed the susceptibility of Vero E6, MA-104, SW-13 and HeLa cell lines to infection by SARS-CoV-2. We determined that the Vero E6 and MA-104 cell lines were permissive for SARS-CoV-2 infection. Initial CPE formation in Vero E6 and MA-104 cell lines infected with SARS-CoV-2 was observed as early as at 24 h post-infection (Figs 1B and 3B, respectively). Immunoblotting analysis also confirmed that only Vero E6 and MA-104 cell lines infected with SARS-CoV-2 showed the expression of the virus specific proteins expression (Fig 5). In addition to Vero E6 cells, the MA104 cell line pertaining to its suitability for SARS-CoV-2 proliferation and facilitate further study of SARS-CoV-2. Our results are in agreement with the previous reports showing that SARS-CoV replicated efficiently and caused CPE in Vero and MA-104 cell lines [44–47].

In order to evaluate the viral growth kinetics of SARS-CoV-2, the Vero E6 and MA-104 cell lines were infected with at an MOI of 0.1. Viral replication was assessed at different time points (6, 12, 18, 24, 48 and 72 h post-infection). We used FFA for all virus titration experiments in this study, since FFA is independent of cell death. SARS-CoV-2 replicated with a similar kinetics in Vero E6 and MA-104 cells, but the MA-104 cells supported replication of SARS-CoV-2 slightly less well than the Vero E6 cells (Fig 6A). Viral replication could be detected at 12 h post-infection, and continued to increase gradually, peaking at 48 h post-infection (Fig 6A and 6B). The decline in virus titer at 72 h post-infection which might be due to death of the infected cells or to the cell lysis (Fig 6A). Our data are in agreement with that of Harcourt et.al., in which SARS-CoV-2 replicated rapidly in Vero cells after an initial eclipse phase and increased gradually, peaking at 48 h post-infection [42].

Lastly, we have compared the whole genome of hCoV-19/Turkey/ERAGEM-001/2020 with a dataset of 3970 available SARS-CoV-2 complete genomes from different countries retrieved from GISAID. The hCoV-19/Turkey/ERAGEM-001/2020 strain was closely clustered with other strains primarily from Australia, Canada, England, Iran, and Kuwait. The reason for the high fraction of Australian cases in this cluster was possibly due to the high number of submitted sequences, as Australia was the third country that had the most sequences submitted to GISAID by April 2020. The cases in the nearby clusters were reported to have a travel history to Iran and shared the common unique nucleotide substitutions, G1397A, T28688C, and G29742T, which were also found in our strain and partially in several Iranian cases [30]. The common key mutations and similar travel histories of the closely clustered cases may indicate possible links between our case and the Iranian epidemic. Interestingly, hCoV-19/Turkey/ERAGEM-001/2020 has the G23876A mutation on the S gene, which is not found in any other full genome sequences in GISAID, including the Australian cluster. The lack of some variants (G11083T, G23876A, and C29563T) in the previously sequenced Turkey strains may also show multiple introductions of strains in the early stages of the epidemic. In this regard, the potential limitation of this finding is the limited number of sequences available for analysis. Further studies with more sequences are needed to determine the distribution of this variant in Turkey and tracing the origin of this strain.

## Conclusions

We have describe the successful isolation of SARS-CoV-2 from a patient in Turkey with confirmed COVID-19. We determined that the Vero E6 and MA-104 cell lines might be a good choice of cell culture model for SARS-CoV-2 that supports viral replication kinetics, development of CPE and subsequent cell death. We also showed that hCoV-19/Turkey/ERAGEM-001/2020 was closely clustered with other strains primarily from Australia, Canada, England, Iran, and Kuwait and that the cases in the nearby clusters were reported to have a travel history to Iran and to share the common unique nucleotide substitutions.

## Supporting information

S1 Raw images (DOCX)
