# Supplementary material for: Isolation and characterization of severe acute respiratory syndrome coronavirus 2 in Turkey
Source: PLoS One. 2020 Sep 16;15(9):e0238614. doi: 10.1371/journal.pone.0238614 (PMC7494126; doi:10.1371/journal.pone.0238614)
Supplement: S1 Raw images — (DOCX) [file pone.0238614.s001.docx]

 **Fig 5**





**Fig 5:** **Western blot assay to examine SARS-CoV-2 proteins production.** The hCoV-19/Turkey/ERAGEM-001/2020 strain was used to infect the cell lines at an MOI of 0.5. The whole cell lysate samples were collected at 24 h post-infection. The Western blot assay was performed to examine the production of viral proteins using a rabbit polyclonal antibody to the SARS-CoV-2 spike glycoprotein (S) (1/1000) (Abcam; ab272504) and a human antibody to the SARS-CoV-2 nucleocapsid protein (NP) (1:2500) (GenScript; HC2003). The membrane was reacted with the ECL substrate solution (Pierce ECL, USA). The membrane was exposed to an autoradiograph film (KODAK X-OMAT, Sigma Germany), and was developed using a Kodak developer (X-OMAT 1000A, Sigma Germany). The arrows indicate that the bands at approximately 180 kDa (Fig 5A) and 48 kDa (Fig 5B) represent S and NP, respectively.


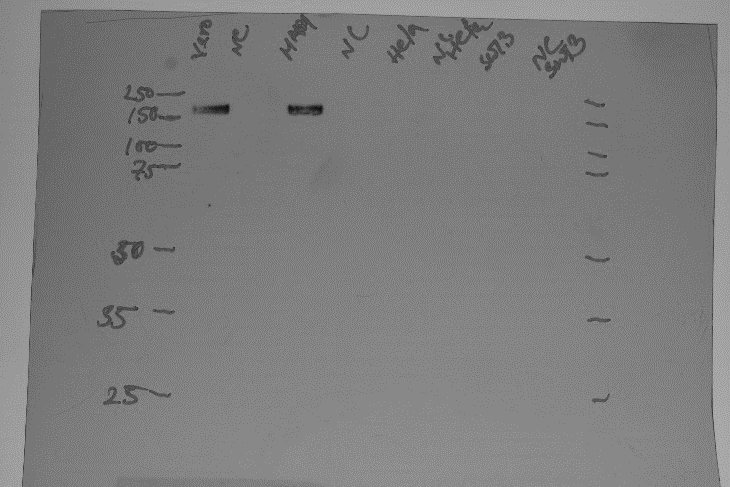
**Uncropped figure**


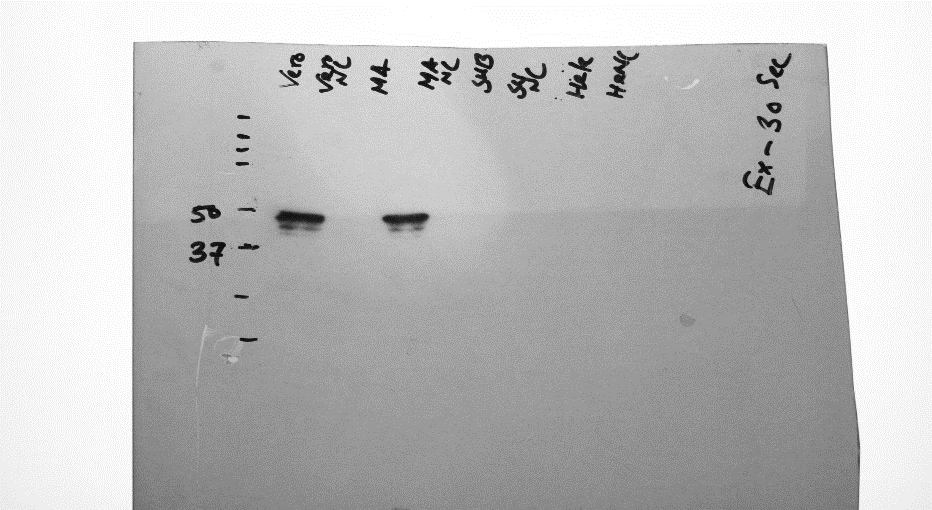


S NP


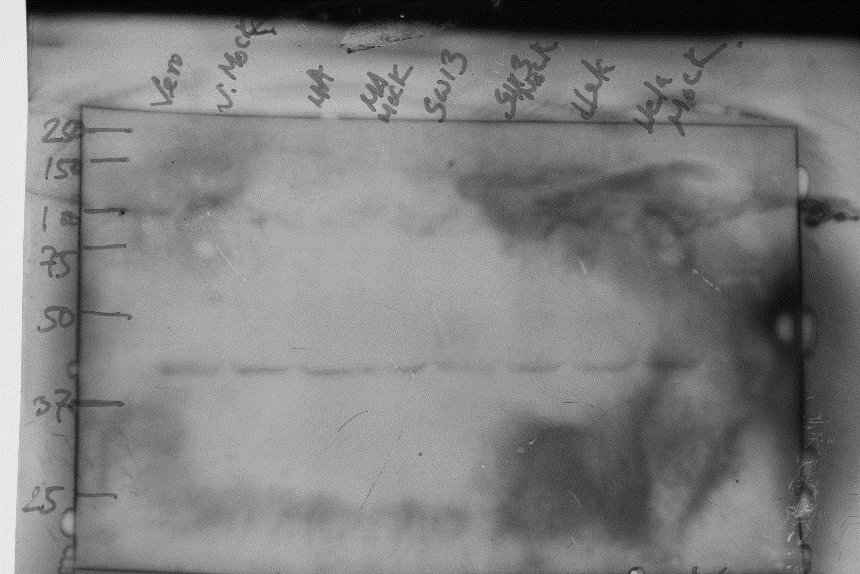

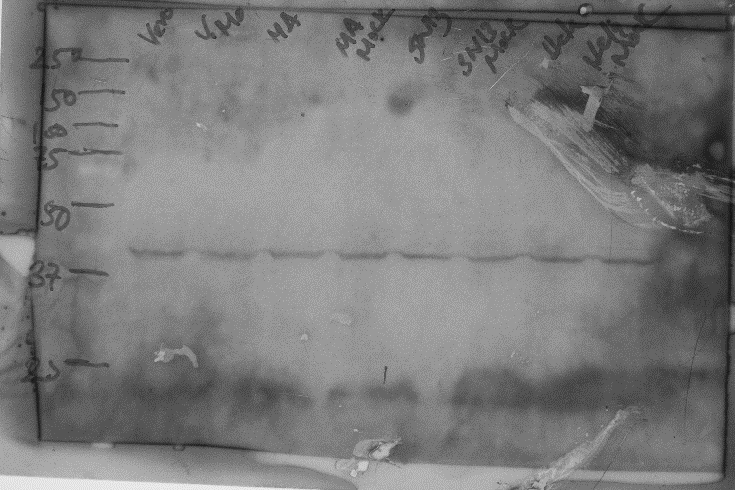


Beta actin Beta actin
